# Post-diagnostic health behaviour scores and risk of prostate cancer progression and mortality

**DOI:** 10.1038/s41416-023-02283-1

**Published:** 2023-05-22

**Authors:** Crystal S. Langlais, Rebecca E. Graff, Erin L. Van Blarigan, John M. Neuhaus, Janet E. Cowan, Jeanette M. Broering, Peter Carroll, Stacey A. Kenfield, June M. Chan

**Affiliations:** 1 Department of Epidemiology and Biostatistics, University of California, San Francisco, San Francisco, CA USA; 2 Helen Diller Family Comprehensive Cancer Center, University of California, San Francisco, San Francisco, CA USA; 3 Department of Urology, University of California, San Francisco, San Francisco, CA USA; 4 Present Address: IQVIA, Durham, NC USA

**Keywords:** Prostate cancer, Risk factors

## Abstract

**Background:**

Individual behaviours are associated with prostate cancer (PC) progression. Behavioural scores, comprised of multiple risk factors, allow assessment of the combined impact of multiple behaviours.

**Methods:**

We examined the association between six a priori scores and risk of PC progression and mortality among 2156 men with PC in the Cancer of the Prostate Strategic Urologic Research Endeavor (CaPSURE) cohort: two scores developed based on the PC survivorship literature (‘2021 Score [+ Diet]’); a score developed based on pre-diagnostic PC literature (‘2015 Score’); and three scores based on US recommendations for cancer prevention (‘WCRF/AICR Score’) and survival (‘ACS Score [+ Alcohol]’). Hazard ratios (HRs) and 95% confidence intervals (CIs) were estimated for progression and PC mortality via parametric survival models (interval censoring) and Cox models, respectively.

**Results:**

Over a median (IQR) of 6.4 (1.3, 13.7) years, we observed 192 progression and 73 PC mortality events. Higher (i.e., healthier) 2021 Score + Diet and WCRF/AICR Scores were inversely associated with risk of PC progression (2021 + Diet: HR_continuous_ = 0.76, 95% CI: 0.63–0.90. WCRF/AICR: HR_continuous_ = 0.83, 95% CI: 0.67–1.02) and mortality (2021 + Diet: HR_continuous_ = 0.65, 95% CI: 0.45–0.93. WCRF/AICR: HR_continuous_ = 0.71; 95% CI: 0.57–0.89). The ACS Score + Alcohol was only associated with progression (HR_continuous_ = 0.89, 95% CI: 0.81–0.98) while the 2021 Score was only associated with PC mortality (HR_continuous_ = 0.62, 95% CI: 0.45–0.85). The 2015 was not associated with PC progression or mortality.

**Conclusion:**

Findings strengthen the evidence that behavioural modifications following a prostate cancer diagnosis may improve clinical outcomes.

## Background

Prostate cancer is the most commonly diagnosed cancer among men in the United States (US), with 248,530 new cases expected to have occurred in 2021 [1, 2]. Currently, there are over 3.6 million prostate cancer survivors in the US [3]. Though the 5-year survival rate for prostate cancer approaches 100%, there remains uncertainty regarding which cancers will eventually progress, and prostate cancer remains the second leading cause of cancer death among US men [2, 4, 5]. To inform interventions and mitigate risk of progression and prostate cancer-specific mortality (PCSM) for the large population of men living with the disease, there is a need to better understand how behavioural factors *after* diagnosis influence disease progression.

Several studies have linked modifiable risk factors with prostate cancer progression and PCSM [6]. However, prior reports have predominantly focused on individual exposures, which do not fully reflect the complex relationships among multiple diet and other behavioural factors [7, 8]. For example, physical activity may offset some of the negative effects of unhealthy dietary choices [9]. Therefore, scores that reflect multiple behavioural factors may be more strongly associated with outcomes among men with prostate cancer than individual health habits.

Our team previously conducted an extensive review summarising the literature on post-diagnostic behaviours and prostate cancer progression and PCSM [6]. Using that report, we developed a prostate cancer-specific behavioural scores (“2021 Score [+ Diet]”). Here, we examine the association of these scores in relation to risk of progression and PCSM among men with non-metastatic prostate cancer in the Cancer of the Prostate Strategic Urologic Research Endeavor (CaPSURE) cohort. Further, for completeness and comparability with other studies, we evaluated associations of four other scores developed to inform the risk of cancer onset or progression to understand if adherence to general cancer prevention or survivorship guidelines may improve outcomes following a prostate cancer diagnosis. One of these scores was developed by members of our team to predict the risk of developing incident lethal prostate cancer based on pre-diagnostic behaviours (“2015 Score”) [10]. It is distinct from the 2021 Scores focused on post-diagnostic behaviours as the known behavioural risk factors for prostate cancer risk and progression differ. The other three are operationalized versions of the American Cancer Society (ACS) cancer survivorship recommendations [11, 12] and the World Cancer Research Fund (WCRF)/American Institute for Cancer Research (AICR) cancer prevention recommendations [13, 14]. We hypothesised that men with healthier lifestyles (i.e., higher scores) would have lower risk of disease progression and mortality.

## Methods

### Study sample

CaPSURE is a longitudinal observational cohort of 15,310 men with biopsy-proven prostate cancer. Men diagnosed between 1999 and 2018 at any of 43 participating urology practices across the US were eligible. Participating urologists provided data on clinical and pathological features, treatments, and clinical follow-up. Additional details on CaPSURE are reported elsewhere [15]. The study was conducted in accordance with the Belmont Report and U.S. Common Rule under local Institutional Review Board approval, with all participants providing written informed consent.

The CaPSURE Diet and Lifestyle (CDL) sub-study—consisting of a comprehensive lifestyle questionnaire and full-length food frequency questionnaire (FFQ)—was administered at three time points between 2004 and 2016; a total of 2891 men participated in at least one administration. For the subset of men who completed more than one questionnaire (*n* = 443), only the first administration (closest to diagnosis date) was used. We excluded men with last clinical follow-up or documented progression prior to completing their first CDL questionnaire (*n* = 551). Consistent with the recommended approach to address implausible energy intakes [16], we excluded men with extreme (<800 kcal/day or >4200 kcal/day) or unknown caloric intake (*n* = 153) and/or missing ≥70 FFQ items (*n* = 20). Finally, we excluded men without a discernable clinical T-stage (*n* = 100) or with a clinical T-stage >T3a (*n* = 8) and those with death from unknown cause (*n* = 3). These exclusions left us with a sample size of 2056 men for our primary analyses of prostate cancer progression. Following the exclusion of men with documented progression prior to completing the first questionnaire, the subsequent exclusions resulted in the loss of 23 events, 2 of which were PCSM. For PCSM analyses, men who were excluded due to documented progression prior to completion of the CDL questionnaire were included—death could not occur prior to completing the questionnaire—resulting in a sample size of 2447 men.

### Diet and lifestyle questionnaire

Dietary intake was self-reported on a validated [17–19] semiquantitative FFQ, wherein men reported how frequently they consumed a standard unit or portion size of approximately 140 different items. The nine frequency options ranged from never or less than once per month to six or more times per day. FFQ data were sent to the Nutrition Department at the Harvard T.H. Chan School of Public Health, which calculated total intake of nutrients, including total caloric intake and grams of whole grains, fibre, and alcohol. Nutrient intake was calculated by multiplying the nutrient value in the specified portion size of each item on the FFQ by its frequency of intake and then summing across all items. Nutrient values were obtained from the US Department of Agriculture databases [20] supplemented with other sources.

The survey asked men if they had smoked 20 packs of cigarettes or more in their lifetime. If they responded “yes”, they were asked to report additional details regarding their smoking history. Men who responded “no” were considered never smokers.

Men completed a validated physical activity questionnaire which asked them to report their average weekly time spent doing nine types of aerobic and resistance training activities over the prior year [21]. Ten frequency options could be selected, ranging from 0 minutes to 11 or more hours per week. Participants were also asked about their regular walking pace and ability/frequency of climbing stairs.

Other information collected on the survey included height and weight [used to calculate body mass index (BMI; kg/m^2^)]; education level; a brief medical history, including family history of prostate cancer; and a detailed history of the use of vitamins and supplements.

### Behavioural scores

Six a priori scores were evaluated, as described below. All scores were oriented such that increasing values reflected healthier behaviours. Please see Supplemental Tables S1 and S2 for additional details.

#### 2021 score

The 2021 (post-diagnostic) Score was based on an extensive literature review conducted in 2021, summarising behaviours following a prostate cancer diagnosis associated with risk of recurrence, progression, and/or PCSM [6]. To determine the factors for inclusion in the score, we searched PubMed using the terms “prostate cancer” and “progression or mortality” in combination with terms describing individual lifestyle factors. Factors considered for the score included those that (1) exhibited a statistically significant association with metastases or PCSM in at least one study and (2) were corroborated by at least one additional study with an association in the same direction, whether or not statistically significant. In total, we identified seven such factors—smoking status [22–31]; BMI [32–48]; physical activity [49–53]; and intake of saturated fat [54–56], whole milk [57, 58], wine [59, 60], and processed meat [61, 62]. The three non-dietary factors demonstrated the strongest evidence in the literature review. We examined two versions of the 2021 Score, one without (“2021 Score”) and one with (“2021 Score + Diet”) the dietary components. The points per behaviour component ranged from 0 to 1 (see Supplement Table S1), with the points for the four dietary components (whole milk, alcohol, red and processed meat, saturated fat) averaged using the arithmetic mean to create a single dietary sub-score ranging from 0 to 1. This approach was consistent with the operationalization of the ACS recommendations into the ACS Score [13]. The point values were based on where the risk associated with prostate cancer outcomes appeared to change in the literature. The points for each component were summed to create the total 2021 Score (range: 0–3) and 2021 Score + Diet (range: 0–4) for each participant.

#### 2015 score

Our team previously developed the 2015 (pre-diagnostic) Score to identify the risk of developing lethal prostate cancer among healthy men, based on the evidence available circa 2014 [10]. The six components—smoking status, BMI, physical activity, fatty fish intake, tomato intake, and processed red meat intake—were each scored as 0 or 1 based on cut-points associated with risk as reported in the literature at the time of score creation. The sub-scores were then summed to create the total 2015 Score (range: 0–6). Components of the 2015 Score were identified based on the existing literature available in 2015.

#### ACS score

To create a primary and an alternative ACS Score, we expanded on the operationalization of the ACS Nutrition and Physical Activity Guidelines for Cancer Survivors developed by McCullough et al. [13]. Each of the three components—BMI, physical activity, and dietary—were scored from 0 to 2 and then summed to create the primary ACS Score (range: 0–6). The dietary component included total servings and variety of fruits and vegetables, red and processed meat intake, and whole grain intake. We expanded to include strength training when assigning physical activity points, consistent with the guidelines. The “ACS Score + Alcohol” additionally included alcohol intake, scored from 0 to 2 (with the highest score for moderate alcohol intake: >0 to 2 servings/day), reflecting the inclusion of alcohol in the ACS recommendations for cancer prevention but not cancer survival (alternative score range: 0–8).

#### WCRF/AICR score

The WCRF/AICR Cancer Prevention Recommendations were operationalized based on published scoring guidelines [11, 12] and included BMI; physical activity; and intake of alcohol, sugar-sweetened beverages, fruits/vegetables, fibre, red and processed meat, and adapted ultra-processed foods (range: 0–7).

### Outcome

The primary outcome was time to prostate cancer progression, defined as biochemical recurrence, secondary treatment, bone metastases, or PCSM, as applied previously [9, 53, 63]. Given the small number of PCSM events (*n* = 73) in this cohort, PCSM was evaluated as a secondary outcome.

*Biochemical recurrence* was defined as two consecutive prostate-specific antigen (PSA) readings ≥ 0.2 ng/mL following radical prostatectomy or a rise of 2.0 ng/mL above post-radiation nadir on two consecutive PSA readings; the date of recurrence was recorded as the date of the second elevated PSA. *Secondary treatment* was defined as any treatment started at least 6 months following primary treatment. *Bone metastases* included prostate cancer progression to bone, advancement to TNM stage M1b, a positive bone scan, and radiation to treat bone metastases. Cause of death was determined by the registry data coordinating centre and through confirmation by either the vital statistics official death certificate from the state in which the death occurred or by the National Centre for Health Statistics National Death Index [64]. Deaths were attributed to prostate cancer if the death certificate included ICD-9 code 185 [(metastatic) malignant neoplasm of prostate] as the primary or secondary cause of death.

Time to progression was measured from completion date of the CDL questionnaire to the date of progression (first event of biochemical recurrence, secondary treatment, bone metastases, or PCSM). For men with documented non-PCSM progression (i.e., recurrence, secondary treatment, or bone metastasis failure events), the censoring interval (i.e., window in which the event occurred) was bound by the last normal clinical visit (left limit) and the clinical visit documenting evidence of progression (right limit). For men who died from prostate cancer, the left and right limit were both date of death. Men without documented progression or PCSM were censored at their last date of follow-up or death (other cause); thus, the left limit of their censoring interval was defined by the last clinical follow-up date or date of death (non-PCSM), respectively, and the right limit was undefined (i.e., censored). Clinical follow-up was last consistently assessed across all CaPSURE sites on January 31, 2019; 26 men had a last known clinical follow-up date beyond this date and were administratively censored on that date.

### Statistical analysis

Parametric survival models with a Weibull distribution were used to accommodate interval censoring associated with uncertainty in actual date of prostate cancer progression [65]. Because the date of death is known for PCSM (i.e., interval censoring was not an issue), we utilised Cox proportional hazards models rather than parametric survival methods when assessing the PCSM outcome. Proportional hazards assumptions were assessed visually by plotting the scaled Schoenfeld residuals against follow-up time.

We fit survival models using both continuous scores (per 1-unit change) and tertiles of scores. All models were clustered by CaPSURE clinical site with robust standard errors used to calculate confidence intervals (CI). Simple models were adjusted for time between diagnosis and participants’ first CDL questionnaire (continuous) and age at diagnosis. A directed acyclic graph (DAG) was developed to reflect our understanding of the complex relationship of interest; variables identified in the DAG were included as covariates in the fully adjusted models, as appropriate [66, 67]. Fully adjusted models were additionally adjusted for clinical T-stage (T1, T2, T3a), Gleason score (<7, 7, >7), diagnostic PSA level (≤6 ng/mL, >6 to 10 ng/mL, >10 ng/mL), primary treatment (radical prostatectomy, radiation, hormonal therapy, watchful waiting/active surveillance, other), family history of prostate cancer in a brother or father (yes, no), self-identified and physician-reported race (white, non-white), selenium supplement use (non-user; <140 µg/day; ≥140 µg/day; user with unknown daily dosage), total caloric intake (continuous, kcal/d), and the following variables if not part of the score of interest: whole milk intake (≤4 servings/week, >4 servings/week), wine intake (3–14 servings/week, <3 or ≥14 servings/week), alcohol intake (non-drinker, >0–2 servings/day, >2 servings/day), red and processed meat intake (quartiles), tomato intake (continuous, servings/day), dark fish intake (continuous, servings/day), and smoking (never, quit ≥10 years prior, quit <10 years prior, current). We further considered adjustment for comorbidities (diabetes, stroke, prior myocardial infarction, or other heart disease; yes/no) but the magnitudes of the estimates changed very little with adjustment, so these variables were not included in the final models.

We assessed potential interaction between each of the scores and age at diagnosis (<65 years, ≥65 years) and, separately, stage at diagnosis (T1, T2–T3a) by adding interaction terms with the scores in the models and using Wald tests. Given statistically non-significant Wald tests and small magnitudes of estimated interaction regression coefficients, interaction terms were not included in the final models. We examined goodness-of-fit of the survival models using Cox–Snell residual plots. Across all scores, goodness-of-fit was best in the fully adjusted models, with decreasing fit in the tails. Fully adjusted models for progression were also run using exponential distributions, which produced Cox–Snell residual plots that demonstrated poorer fit than Weibull models and thus were not reported.

### Sensitivity analyses

First, we were concerned about confounding due to PSA surveillance after diagnosis (i.e., men with healthier behaviours may be more likely to be monitored via PSA tests, potentially creating a positive correlation between healthy lifestyle habits and risk of progression). To address this, Poisson regression was utilised to compare the number of PSA visits to tertile of each of the six scores, with the lowest tertile (i.e., the least healthy group) as the reference. Total follow-up time was used as an offset in these models.

Second, whereas our primary analyses used time of the CDL questionnaire completion as time zero—which necessitates excluding men who experienced an event prior to the survey—sensitivity analyses re-assigned time zero as time of diagnosis. These analyses assumed that the responses on the CDL questionnaire were consistent with what would have been measured at the date of diagnosis. Men excluded from our primary analyses due to documented progression prior to CDL questionnaire were included in these sensitivity analyses, resulting in an analytic sample of 2447 men. For this approach, we first assessed whether there was an interaction between year of diagnosis and each of the behavioural scores by adding an interaction term with the scores in the models and using Wald tests; no evidence of interaction was found.

Third, we were interested in understanding how competing events (i.e., deaths due to causes other than prostate cancer) impacted our primary results. Methods to address competing events in the presence of interval censoring are not readily available or accessible. Thus, we ran Cox proportional hazards models for progression and compared these results to Fine-Gray analyses accounting for other deaths as a competing risk. Proportional hazards assumptions were assessed visually by plotting the scaled Schoenfeld residuals against follow-up time.

Lastly, missingness in the covariates resulted in a loss of events in our fully adjusted models. Specifically, men with missing data for any of the score components were excluded from the primary analysis for that score: *n* = 60 for 2021 Score, *n* = 60 for the 2021 Score with Diet, *n* = 83 for 2015 Score, *n* = 40 for ACS Score, *n* = 70 for the ACS Score with Alcohol, and *n* = 43 for WCRF/AICR Score. To understand the impact of this missingness on our primary results, we performed sensitivity analyses utilising multiple imputation to handle missing data [68], which assumes that data are missing at random. We assessed the plausibility of this assumption by summarising participant characteristics by missingness status for each of the six scores. We performed multiple imputation via chained equations using the *chained* command in Stata to first generate 25 imputed datasets. We then fit survival models across all 25 imputed datasets and pooled the results using Rubin’s Rules [69]. Our imputed models included fully observed variables (CaPSURE clinical site, age at diagnosis, BMI, days of follow-up, total energy intake, tomato intake, days from CDL return to the left interval of follow-up time, race, diagnostic T-stage, and family history of prostate cancer) and variables with incomplete values (diagnostic PSA and Gleason score; total alcohol, whole milk, dark fish, total wine, and red and processed meat intake; each of the scores; smoking status; and primary treatment).

All statistical analyses were performed using Stata version 17 (StataCorp, College Station, TX) using a two-sided alpha level of 0.05 to assess statistical significance.

## Results

In our main analyses, the 2056 men who met inclusion criteria were followed for a median of 6.4 years (IQR: 1.3, 12.7) after completing the CDL questionnaire, for a total of 13,102 person-years. During the follow-up period, 192 had documented progression, including 168 (88%) with biochemical recurrence, 7 (4%) with bone metastases, and 17 (9%) deaths related to prostate cancer as the first recorded event (there were 73 PCSM events in total). There were 384 all-cause deaths during the observation period. Baseline characteristics by tertile of each of the four primary scores are shown in Table 1. Most participants identified as white race with a diagnostic T-stage of 1 and Gleason grade <7 and underwent radical prostatectomy as their primary treatment. Characteristics were balanced across tertiles of the scores.

**Table 1:** Patient and clinical characteristics of men diagnosed with non-metastatic prostate cancer by tertile of health behaviour scores.

	2021 Score^a^			2015 Score^a^			ACS Score^a^			WCRF/AICR Score^a^		
*N*^b^:	1996			1973			2016			2013		
Tertile:	1st	2nd	3rd	1st	2nd	3rd	1st	2nd	3rd	1st	2nd	3rd
Point range:	0–2	2.5	3	0–3	4	5–6	0–2	2.5–3	3.5–6	0.75–3.25	3.5–4	4.25–7
*n*	1033	640	323	1259	475	239	710	610	696	805	560	648
Age^c^ (years), mean (SD)	64.5 (7.9)	64.0 (7.9)	64.7 (8.3)	64.6 (7.9)	64.2 (7.9)	63.3 (8.1)	64.0 (7.8)	64.8 (8.0)	64.3 (8.1)	64.4 (7.8)	64.4 (7.9)	64.3 (8.2)
White, *n* (%)	979 (95)	609 (95)	310 (96)	1202 (95)	455 (96)	220 (92)	672 (95)	585 (96)	660 (95)	764 (95)	531 (95)	620 (96)
T-Stage^c^, *n* (%)												
T1	598 (58)	367 (57)	184 (57)	697 (55)	284 (60)	156 (65)	407 (57)	336 (55)	416 (60)	483 (60)	324 (58)	350 (54)
T2	426 (41)	268 (42)	134 (41)	550 (44)	186 (39)	81 (34)	297 (42)	270 (44)	271 (39)	315 (39)	230 (41)	292 (45)
T3a	9 (1)	5 (1)	5 (2)	12 (1)	5 (1)	2 (1)	6 (1)	4 (1)	9 (1)	7 (1)	6 (1)	6 (1)
Gleason^c^, *n* (%)												
<7	680 (66)	435 (69)	223 (69)	816 (65)	332 (70)	174 (73)	472 (67)	404 (67)	470 (68)	518 (65)	388 (70)	440 (68)
7	278 (27)	164 (26)	75 (23)	343 (28)	115 (24)	51 (21)	178 (25)	168 (28)	182 (26)	219 (28)	138 (25)	167 (26)
>7	67 (7)	35 (6)	23 (7)	87 (7)	25 (5)	13 (5)	55 (8)	31 (5)	40 (6)	59 (7)	30 (5)	37 (6)
PSA^c^ (ng/ml), median (IQR)	5.6 (4.4, 8.0)	5.5 (4.3, 7.6)	5.3 (4.3, 7.5)	5.6 (4.4, 8.0)	5.5 (4.5, 7.6)	5.2 (4.1, 7.6)	5.6 (4.4, 8.0)	5.6 (4.4, 7.7)	5.4 (4.3, 7.8)	5.7 (4.4, 8.0)	5.5 (4.3, 7.7)	5.4 (4.3, 7.7)
Primary treatment, *n* (%)												
Radical prostatectomy	604 (60)	402 (65)	210 (67)	769 (62)	282 (61)	153 (67)	407 (59)	376 (63)	448 (66)	473 (60)	340 (62)	413 (66)
AS/WW	56 (6)	39 (6)	23 (7)	64 (5)	33 (7)	19 (8)	32 (5)	37 (6)	49 (7)	40 (5)	36 (7)	42 (7)
RT/brachytherapy	245 (24)	129 (21)	58 (19)	281 (23)	101 (22)	43 (19)	175 (25)	129 (22)	131 (19)	189 (24)	123 (22)	124 (20)
Hormone therapy	61 (6)	26 (4)	11 (4)	61 (5)	25 (5)	9 (4)	40 (6)	40 (7)	21 (3)	46 (6)	30 (5)	25 (4)
Other	47 (5)	21 (3)	11 (4)	57 (5)	19 (4)	4 (2)	37 (5)	14 (2)	27 (4)	35 (4)	22 (4)	22 (4)
Family history of PC, *n* (%)	186 (18)	141 (22)	79 (24)	246 (20)	105 (22)	51 (21)	143 (20)	115 (19)	151 (22)	171 (21)	121 (22)	116 (18)

*ACS* American Cancer Society, *AICR* American Institute for Cancer Research, *AS/WW* active surveillance/watchful waiting, *aUPFs* adapted ultraprocessedfoods, *BMI* body mass index, *IQR* interquartile range, *PC* prostate cancer, *RT* radiation therapy, *SD* standard deviation, *WCRF* World Cancer Research Fund.

^a^2021 Score included smoking, BMI, and physical activity. 2015 Score included smoking, BMI, physical activity, fatty fish intake, tomato intake, and processed red meat intake. ACS Score included BMI, physical activity, whole fruit and vegetables intake, red and processed meat intake, and whole grain intake. WCRF/AICR Score included BMI, physical activity, alcohol intake, sugar-sweetened beverage intake, percentage of calories from aUPFs, fiber intake, whole fruit and vegetable intake, and red and processed meat intake.

^b^Missingness on score components resulted in missing scores: *n* = 60 for 2021 Score, *n* = 83 for 2015 Score, *n* = 40 for ACS Score, and *n* = 43 for AICR Score.

^c^At prostate cancer diagnosis.

### Progression

**Fig. 1:**
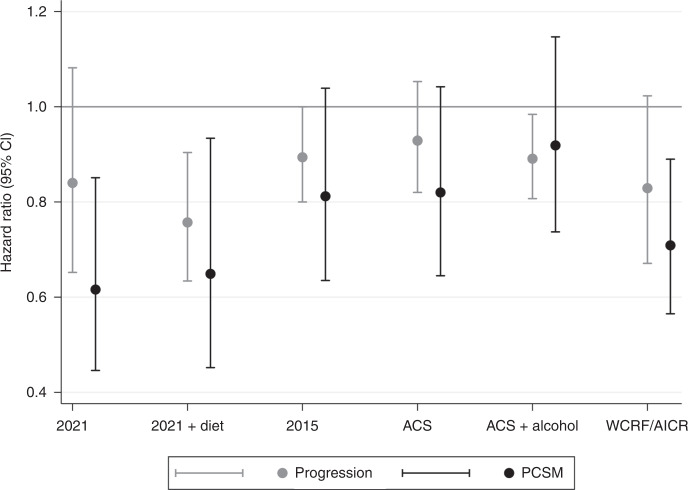
Post-diagnostic health behaviour scores and the risk of prostate cancer progression and mortality among men with non-metastatic prostate cancer. Visual summary of the HR and 95% CI for the risk of progression (light grey) and PCSM (dark grey) per 1-unit increase (i.e, healthier score) in each of the 6 behavioural scores examined.

Those with higher 2021 Scores had a non-statistically significant lower risk of progression (HR_cont_: 0.84, 95% CI: 0.65–1.08); however, in models assessing score tertiles, there was no clear association with progression (HR_2 vs 1_: 0.90, 95% CI: 0.62–1.32; HR_3 vs 1_: 0.79, 95% CI: 0.50–1.24; *P*_trend_ = 0.30). Including dietary factors in the 2021 Score (2021 Score + Diet) strengthened the associations: HR_cont_: 0.76, 95% CI: 0.63–0.90; HR_2 vs 1_: 0.82, 95% CI: 0.62–1.08 and HR_3 vs 1_: 0.67; 95% CI: 0.44–1.02 (*P*_trend_ = 0.06) (Table 2 and Fig. 1).

**Table 2:** Post-diagnostic health behaviour scores and the risk of prostate cancer progression among men with non-metastatic prostate cancer, estimated via parametric (Weibull) survival models.

2021 Score—HR (95% CI)													
	Events	*N*	Continuous			1st Tertile	2nd Tertile			3rd Tertile			*P*_trend_
						(0–2 pts)	(2.5 pts)			(3 pts)			
Simple^a^	188	1996	0.79	(0.66,	0.95)	Ref	0.77	(0.57,	1.04)	0.69	(0.46,	1.04)	0. 07
Fully adjusted^b^	146	1615	0.84	(0.65,	1.08)	Ref	0.90	(0.62,	1.32)	0.79	(0.50,	1.24)	0.30

2021 Score + Diet—HR (95% CI)													
	Events	*N*	Continuous			1st Tertile	2nd Tertile			3rd Tertile			*P*_trend_
						(0.25–2.25 pts)	(2.5–3 pts)			(3.25–4 pts)			
Simple^a^	188	1996	0.78	(0.68,	0.90)	Ref	0.83	(0.67,	1.04)	0.67	(0.46,	0.97)	0.03
Fully adjusted^b^	151	1673	0.76	(0.63,	0.90)	Ref	0.82	(0.62,	1.08)	0.67	(0.44,	1.02)	0.06

2015 Score—HR (95% CI)													
	Events	*N*	Continuous			1st Tertile	2nd Tertile			3rd Tertile			*P*_trend_
						(0–3 pts)	(4 pts)			(5–6 pts)			
Simple^a^	183	1973	0.85	(0.78,	0.94)	Ref	0.82	(0.59,	1.14)	0.56	(0.33,	0.94)	0.03
Fully adjusted^b^	141	1611	0.89	(0.80,	1.00)	Ref	0.90	(0.60,	1.35)	0.57	(0.30,	1.09)	0.09

ACS Score—HR (95% CI)													
	Events	*N*	Continuous			1st Tertile	2nd Tertile			3rd Tertile			*P*_trend_
						(0–2 pts)	(2.5–3 pts)			(3.5–6 pts)			
Simple^a^	188	2016	0.89	(0.80,	0.98)	Ref	0.93	(0.68,	1.28)	0.71	(0.53,	0.94)	0.02
Fully adjusted^b^	146	1614	0.93	(0.82,	1.05)	Ref	1.19	(0.82,	1.71)	0.83	(0.58,	1.18)	0.30

ACS Score + Alcohol—HR (95% CI)													
	Events	*N*	Continuous			1st Tertile	2^nd^ Tertile			3rd Tertile			*P*_trend_
						(0–3.5 pts)	(4–5 pts)			(5.5–8 pts)			
Simple^a^	182	1986	0.88	(0.80,	0.96)	Ref	0.77	(0.56,	1.05)	0.49	(0.29,	0.83)	0.008
Fully adjusted^b^	146	1614	0.89	(0.81,	0.98)	Ref	0.97	(0.71,	1.32)	0.48	(0.28,	0.82)	0.007

WCRF/AICR Score—HR (95% CI)													
	Events	*N*	Continuous			1st Tertile	2nd Tertile			3rd Tertile			*P*_trend_
						(0.75–3.25 pts)	(3.5–4 pts)			(4.25–7 pts)			
Simple^a^	188	2013	0.85	(0.72,	1.01)	Ref	0.79	(0.52,	1.20)	0.63	(0.43,	0.91)	0.01
Fully adjusted^b^	146	1618	0.83	(0.67,	1.02)	Ref	0.89	(0.51,	1.55)	0.60	(0.36,	1.01)	0.05

*ACS* American Cancer Society, *Adj* adjusted, *AICR* American Institute for Cancer Research, *CI* confidence interval, *HR* hazard ratio, *pts* points, *WCRF* World Cancer Research Fund, *wk* week.

^a^Simple models were adjusted for time between diagnosis and date of first CDL questionnaire (continuous), age at diagnosis (continuous) and CaPSURE clinical site.

^b^Fully adjusted models were additionally adjusted for clinical T-stage (T1, T2, T3), Gleason score (<7, 7, >7), diagnostic PSA value (≤6, >6–10, >10–20), primary treatment (radical prostatectomy, active surveillance/watchful waiting, radiotherapy/brachytherapy, hormone therapy, other), family history of prostate cancer in brother of father (yes, no), race (white, non-white), total caloric intake (continuous), plus the following variables (if not part of the score): whole milk intake (≤4 servings/wk, >4 servings/wk), wine intake (3–14 servings/wk, <3 or >14 servings/wk), total alcohol intake (non-drinker, >0–2 serving/day, >2 servings/day), red and processed meat intake (quartiles), tomato intake (continuous), dark meat fish intake (continuous), selenium supplement use (non-user, <140 µg/day, ≥140 µg/day, user with unknown daily dosage), smoking (never, quit ≥10 years prior, quit <10 years prior, current).

Neither the 2015 Score (HR_cont_: 0.89, 95% CI: 0.80–1.00; HR_2 vs 1_: 0.90, 95% CI: 0.60–1.35; HR_3 vs 1_: 0.57, 95% CI: 0.30–1.09; *P*_trend_ = 0.091) nor the ACS Score were associated with risk of prostate cancer progression (HR_cont_: 0.93; 95% CI: 0.82–1.05; HR_2 vs 1_: 1.19, 95% CI: 0.82–1.71; HR_3 vs 1_: 0.83; 95% CI: 0.58–1.18; *P*_trend_ = 0.30). The ACS Score + Alcohol, however, demonstrated evidence of an inverse association with risk of progression (HR_cont_: 0.89, 95% CI: 0.81–0.98; HR_2 vs 1_: 0.97, 95% CI: 0.71–1.32; HR_3 vs 1_: 0.48, 95% CI: 0.28-0.82; *P*_trend_ = 0.007). The WCRF/AICR Score was also inversely associated with risk of prostate cancer progression (HR_cont_: 0.83, 95% CI: 0.67–1.02; HR_2 vs 1_: 0.89, 95% CI; 0.51–1.55; HR_3 vs 1_: 0.60, 95% CI: 0.36–1.01; *P*_trend_ = 0.05) (Table 2 and Fig. 1).

### Prostate cancer-specific mortality

The 2021 Score was statistically significantly associated with a lower risk of PCSM (HR_cont_: 0.62, 95% CI: 0.45–0.85). However, in models assessing score tertiles, there was no clear association with PCSM (HR_2 vs 1_: 0.50, 95% CI: 0.23–1.06; HR_3 vs 1_: 0.71, 95% CI: 0.38-1.33; *P*_trend_ = 0.28). When dietary factors were included (2021 Score + Diet), associations with PCSM were statistically significant in continuous (HR: 0.65; 95% CI: 0.45, 0.93) and tertile models, showing a 59% reduced risk of PCSM among those with the highest versus the lowest tertile of score (HR_3 vs 1_: 0.41, 95% CI: 0.20, 0.85; *P*_trend_ = 0.02) (Table 3 and Fig. 1).

**Table 3:** Post-diagnostic health behaviour scores and the risk of prostate cancer mortality among men with non-metastatic prostate cancer, estimated via Cox proportional hazards models^a^.

2021 Score—HR (95% CI)													
	Events	*N*	Continuous			1st Tertile	2nd Tertile			3rd Tertile			*P*_trend_
						(0–2 pts)	(2.5 pts)			(3 pts)			
Simple^b^	69	2369	0.73	(0.52,	1.03)	Ref	0.64	(0.39,	1.04)	0.73	(0.40,	1.35)	0.32
Fully adjusted^c^	51	1877	0.62	(0.45,	0.85)	Ref	0.50	(0.23,	1.06)	0.71	(0.38,	1.33)	0.28

2021 Score with Diet—HR (95% CI)													
	Events	*N*	Continuous			1st Tertile	2nd Tertile			3rd Tertile			*P*_trend_
						(0.25–2.25 pts)	(2.5–3 pts)			(3.25–4 pts)			
Simple^b^	69	2369	0.76	(0.55,	1.04)	Ref	0.64	(0.37,	1.09)	0.59	(0.33,	1.06)	0.078
Fully adjusted^c^	52	1947	0.65	(0.45,	0.93)	Ref	0.55	(0.30,	1.02)	0.41	(0.20,	0.85)	0.02

2015 Score—HR (95% CI)													
	Events	*N*	Continuous			1st Tertile	2nd Tertile			3rd Tertile			*P*_trend_
						(0–3 pts)	(4 pts)			(5–6 pts)			
Simple^b^	65	2336	0.86	(0.71,	1.04)	Ref	0.96	(0.64,	1.43)	0.72	(0.33,	1.57)	0.41
Fully adjusted^c^	49	1874	0.81	(0.63,	1.04)	Ref	0.94	(0.65,	1.37)	0.61	(0.18,	2.03)	0.42

ACS Score—HR (95% CI)													
	Events	*N*	Continuous			1st Tertile	2nd Tertile			3rd Tertile			*P*_trend_
						(0–2 pts)	(2.5–3 pts)			(3.5–6 pts)			
Simple^b^	69	2396	0.84	(0.69,	1.02)	Ref	0.74	(0.42,	1.32)	0.64	(0.35,	1.15)	0.13
Fully adjusted^c^	50	1875	0.82	(0.65,	1.04)	Ref	0.65	(0.36,	1.17)	0.58	(0.29,	1.15)	0.12

ACS Score with alcohol—HR (95% CI)													
	Events	*N*	Continuous			1st Tertile	2nd Tertile			3rd Tertile			*P*_trend_
						(0–3.5 pts)	(4–5 pts)			(5.5–8 pts)			
Simple^b^	68	2359	0.87	(0.74,	1.03)	Ref	0.74	(0.41,	1.33)	0.73	(0.37,	1.42)	0.35
Fully adjusted^c^	50	1875	0.92	(0.74,	1.15)	Ref	1.07	(0.49,	2.37)	0.82	(0.35,	1.93)	0.64

WCRF/AICR Score—HR (95% CI)													
	Events	*N*	Continuous			1st Tertile	2nd Tertile			3rd Tertile			*P*_trend_
						(0.75–3.25 pts)	(3.5–4 pts)			(4.25–7 pts)			
Simple^b^	69	2395	0.80	(0.69,	0.94)	Ref	0.66	(0.37,	1.17)	0.57	(0.38,	0.84)	0.004
Fully adjusted^c^	50	1883	0.71	(0.57,	0.89)	Ref	0.51	(0.25,	1.03)	0.52	(0.33,	0.81)	0.004

*ACS* American Cancer Society, *Adj* adjusted, *AICR* American Institute for Cancer Research, *CI* confidence interval, *HR* hazard ratio, *pts* points, *WCRF* World Cancer Research Fund, *wk* week.

^a^A total of 2447 men met inclusion for PCSM analyses, as men who were excluded from progression analysis due to having a documented progression event prior to questionnaire were included in this analysis.

^b^Simple models were adjusted for time between diagnosis and date of first CDL questionnaire (continuous) and age at diagnosis (continuous).

^c^Fully adjusted models were additionally adjusted for clinical T-stage (T1, T2, T3), Gleason score (<7, 7, >7), diagnostic PSA value (≤6, >6–10, >10–20), primary treatment (radical prostatectomy, active surveillance/watchful waiting, radiotherapy/brachytherapy, hormone therapy, other), family history of prostate cancer in brother of father (yes, no), race (white, non-white), total caloric intake (continuous), plus the following variables (if not part of the score): whole milk intake (≤4 servings/wk, >4 servings/wk), wine intake (3–14 servings/wk, <3 or >14 servings/wk), total alcohol intake (non-drinker, >0–2 serving/day, >2 servings/day), red and processed meat intake (quartiles), tomato intake (continuous), dark meat fish intake (continuous), selenium supplement use (non-user, <140 µg/day, ≥140 µg/day, user with unknown daily dosage), smoking (never, quit ≥10 years prior, quit <10 years prior, current).

There was no association with PCSM for the 2015 (HR_cont_: 0.81, 95% CI: 0.63–1.04), ACS (HR_cont_: 0.82, 95% CI: 0.65–1.04) or ACS + Alcohol (HR_cont_: 0.92, 95% CI: 0.74–1.15) Scores (Table 3 and Fig. 1). The WCRF/AICR Score was inversely associated with risk of PCSM (HR_cont_: 0.71, 95% CI: 0.57–0.89), amounting to a 48% (HR: 0.52, 95% CI: 0.33–0.81; *P*_trend_ = 0.004) lower risk among those with the highest versus lowest tertile of score (Table 3 and Fig. 1).

### Sensitivity analyses

Across all scores, there was no evidence that men with higher behavioural scores presented more frequently for PSA monitoring following a diagnosis (data not shown). In models that imposed date of diagnosis as time zero, the trends were similar across all scores (Supplemental Table S3). The results from Cox proportional hazards models for progression were similar to those from the parametric (Weibull) survival and there was no evidence that competing events impacted the results (Supplemental Table S4). Multiple imputation resulted in 2056 complete records and retention of all 192 events in multivariable models. Across all scores, with the exception of age, characteristics were similar between men with and without missingness, providing some evidence that data were missing at random (Supplemental Table S5). The results following imputation were similar to those obtained from the complete-case analysis. With the larger sample sizes, however, the confidence intervals tightened, resulting in statistically significant estimates across all scores (Supplemental Table S6).

## Discussion

In this prospective study, we examined associations of behavioural risk scores with prostate cancer progression and PCSM among men diagnosed with non-metastatic prostate cancer. For each 1-unit increase (i.e., healthier) in the 2021 Score + Diet and the ACS Score + Alcohol, men had a statistically significant 24% and 11% lower risk of progression, respectively. The WCRF/AICR Score was also associated with a (statistically non-significant) reduced risk of progression, demonstrating a 17% lower risk of progression per point increase. Men in the highest tertile of the 2021 Score + Diet and WCRF/AICR Score had a 59% and 48%, respectively, lower risk of dying from prostate cancer compared to those in the lowest tertile.

The difference in associations observed between the two outcomes may reflect different mechanisms driving recurrence versus PCSM. Indeed, 94% of men with biochemical recurrence in this cohort did not die from prostate cancer during study follow-up. Another explanation is confounding: healthier men may present more often for PSA monitoring and thus be more likely to have biochemical recurrence detected which may spuriously attenuate associations. We attempted to evaluate whether such confounding bias impacted our results and did not observe different screening behaviours based on score levels. Nevertheless, we cannot rule out confounding.

Importantly, components varied across behavioural scores and were used differently within scores. For example, the ACS Score + Alcohol assigned the highest (i.e., healthiest) points for moderate alcohol intake, whereas WCRF/AICR Score preserved highest points for no alcohol intake. The 2021 Score + Diet only included moderate consumption of wine in its highest point. Aligned with ACS recommendations, the decision to consume alcohol should be made on an individual basis with a patient’s provider [70].

The 2015 Score was developed based on the literature describing the risk of developing lethal prostate cancer among disease-free men; [10] our team previously reported that this score was associated with a 68% lower risk (5–6 points vs. 0–1 points) of developing lethal prostate cancer among disease-free men [10]. However, our results, in combination with existing evidence, suggest that behavioural factors associated with *developing* prostate cancer may differ from those associated with *progression and mortality following a diagnosis* [71, 72].

While not all statistically significantly, all scores were *inversely* associated with both outcomes, supporting our hypothesis that higher scores/healthier lifestyle patterns would be protective for progression and PCSM. Considering similarities and differences in score composition may provide insights for further scientific exploration. For example, while both the 2021 Score + Diet and WCRF/AICR Score had statistically significant inverse associations with PCSM, the 2021 Score + Diet was associated with a slightly larger reduction in risk. This suggests that while general “healthy” diet recommendations for total cancer prevention (e.g., WCRF/AIRC) are good, additional specific guidance (e.g., limited intake of whole milk) for preventing *prostate cancer* death may be warranted. Also, the 2021 Score + Diet was the only score to consider saturated fat intake in addition to specific high-fat foods. This information may guide hypothesis generation regarding biological mechanisms linking health behaviours to PC outcomes. Confirmation of the performance of the 2021 Score + Diet in independent study populations is warranted, and could lead to more tailored recommendations for patients with prostate cancer.

There are several limitations of our analyses to consider. Men in our study predominately identified as white race (95%), were well educated (77% with at least some college), and were insured (97%), meaning these results are not generalisable to all men with prostate cancer. Social determinants of health and their impacts on health and disease status cannot be addressed in this cohort. While we observed some statistically significant inverse associations for PCSM, this was a secondary outcome given the limited number of events. Though we made efforts to address potential biases in this study (e.g., multiple imputation to address missingness, modelling PSA surveillance behaviour as a function of behavioural scores to address confounding issues), these approaches are not without their own assumptions, and thus we cannot rule out bias entirely. Finally, the post-diagnostic literature that drove the creation of the 2021 Score (with and without diet) came from a limited number of study populations, which included CaPSURE [6]. This fact underscores the importance of confirming these findings in other populations.

In conclusion, among men diagnosed with non-metastatic prostate cancer, a behavioural score developed based on the current post-diagnostic literature (2021 Score Including Diet) was associated with a 24% lower risk of progression and 35% lower risk of PCSM per one-unit increase in the score. Men diagnosed with non-metastatic prostate cancer may improve survivorship by adhering to post-diagnostic prostate cancer-specific dietary recommendations—avoiding/limiting the consumption of whole milk, red and processed meats, and saturated fat, while allowing moderate consumption of wine—in addition to the general recommendations to avoid smoking, maintain a healthy body size, and engage in regular physical activity.

## Supplementary information

Supplemental Tables

## Data Availability

Data and code can be made available on request.
