# Supplementary material for: Post-diagnostic health behaviour scores and risk of prostate cancer progression and mortality
Source: Br J Cancer. 2023 May 22;129(2):346–55. doi: 10.1038/s41416-023-02283-1 (PMC10338438; doi:10.1038/s41416-023-02283-1)
Supplement: Supplementary file 1 — Supplemental Tables [file 41416_2023_2283_MOESM1_ESM.docx]

| **Supplemental Table S1. Operationalization of the 2021 Score, 2015 Score, ACS Score, and WCRF/AICR Score** | | | | |
| --- | --- | --- | --- | --- |
| **Score^a^:** | **2021 Score**^b^ | **2015 Score** | **ACS**^c^ | **WCRF/AICR** |
| **Point Range:** | 0-3 (with Diet: 0-4) | 0-6 | 0-6 (with Alcohol: 0-8) | 0-7 |
| Smoking Status^d^ | **1:** never or quit ≥10 years prior  **0.5:** quit <10 years prior  **0:** current | **1:** never or quit ≥10 years prior  **0:** quit <10 years prior or current | N/A | N/A |
| Body Mass Index^e^ | **1:** 18.5 to <25 kg/m^2^  **0.5:** 25 to <30 kg/m^2^  **0:** ≥30 kg/m^2^ | **1:** <30 kg/m^2^  **0:** ≥30 kg/m^2^ | **2:** 18.5 to <25 kg/m^2^  **1:** 25 to <30 kg/m^2^  **0:** ≥30 kg/m^2^ | **1:** 18.5 to <25 kg/m^2^  **0.5:** 25 to <30 kg/m^2^  **0:** <18.5 kg/m^2^ or ≥30 kg/m^2^ |
| Physical Activity | **1:** ≥18 MET-hr/wk total PA  **0.5:** 9 to <18 MET-hr/wk total PA  **0:** <9 MET-hr/wk total PA | **1:** ≥3 hr/wk vigorous PA or ≥7 hr/wk brisk walking  **0:** <3 hr/wk vigorous PA and <7 hr/wk brisk walking | **1:** ≥17.5 MET-hr/wk moderate or vigorous aerobic PA  **0.5:** 8.75 to <17.5 MET-hr/wk moderate or vigorous aerobic PA  **0:** <8.75 MET-hr/wk moderate or vigorous aerobic PA  **1:** ≥40 min/wk strength training  **0.5:** >0-<40 min/wk strength training  **0:** 0 min/wk strength training | **1:** ≥2.5 hr/wk moderate or vigorous PA  **0.5:** 1.25 to <2.5 hr/wk moderate or vigorous PA  **0:** <1.25 hr/wk moderate or vigorous PA |
| Fatty Fish | N/A | **1:** ≥1 serv/wk  **0:** <1 serv/wk | N/A | N/A |
| Whole Milk^f^ | **1:** ≤4 serv/wk  **0:** >4 serv/wk | N/A | N/A | N/A |
| Alcohol^f,g^ | **1:** 3 to 14 serv/wk of wine  **0:** <3 or >14 serv/wk wine | N/A | **2:** >0 to 2 serv/day  **1:** 0 serv/day  **0:** >2 serv/day | **1:** 0 gm/day  **0.5:** >0 to 28 gm/day  **0:** >28 gm/day |
| Sugar-Sweetened Beverages | N/A | N/A | N/A | **1:** 0 gm/day  **0.5:** >0 to 250 gm/day  **0:** >250 gm/day |
| Percentage of Calories from aUPFs | N/A | N/A | N/A | **1:** 1^st^ tertile  **0.5:** 2^nd^ tertile  **0:** 3^rd^ tertile |
| Fiber | N/A | N/A | N/A | **0.5:** ≥30 gm/day  **0.25:** 15 to <30 gm/day  **0:** <15 gm/day |
| Whole Fruits and Vegetables^h^ | N/A | **1:** ≥7 serv/wk tomatoes  **0:** <7 serv/wk tomatoes | **1:** ≥5 serv/day  **0:** <5 serv/day  **2:** 3^rd^ tertile of unique fruits and vegetables consumed  **1:** 2^nd^ tertile of unique fruits and vegetables consumed  **0:** 1^st^ tertile of unique fruits and vegetables consumed | **0.5:** ≥400 gm/day  **0.25:** 200 to <400 gm/day  **0:** <200 gm/day |
| Red and Processed Meat^f, h^ | **1:** <2 serv/wk  **0:** ≥2 serv/wk | **1:** <3 serv/wk processed red meat  **0:** ≥3 serv/wk processed red meat | **3:** 1^st^ quartile  **2:** 2^nd^ quartile  **1:** 3^rd^ quartile  **0:** 4^th^ quartile | **1:** <500 gm/wk red meat and <21 gm/wk processed meat  **0.5:** <500 gm/wk red meat and 21 to <100 gm/wk processed meat  **0:** ≥500 gm/wk red meat or ≥100 gm/wk processed meat |
| Whole Grains^h^ | N/A | N/A | **3:** 4^th^ quartile  **2:** 3^rd^ quartile  **1:** 2^nd^ quartile  **0:** 1^st^ quartile | N/A |
| Saturated Fat^f^ | **1:** <10% of total energy intake  **0:** ≥10% of total energy intake | N/A | N/A | N/A |
| Abbreviations: ACS – American Cancer Society; AICR – American Institute for Cancer Research; aUPFs – adapted ultra-processed foods; gm/day – grams per day; hr – hours; MET – metabolic equivalent of task; PA – physical activity; serv – servings; WCRF – World Cancer Research Fund; wk – week  ^a^ Higher scores implied higher compliance with the recommendations within each score.  ^b^ The 2021 Score did not include dietary components and ranged from 0-3. The 2021 Score + Diet included dietary components and ranged from 0-4.  ^c^ The ACS Score did not include alcohol consumption and ranged from 0-6. The ACS Score + Alcohol included alcohol and ranged from 0-8.  ^d^ Former smokers with unknown time since quitting were excluded.  ^e^ The 2021 Score and ACS Score excluded men with BMI <18.5kg/m^2^.  ^f^ Whole milk, wine, red/processed meat, and saturated fat were only included in the 2021 Score + Diet as the arithmetic mean of their sum.  ^g^ Alcohol was only included in the ACS Score + Alcohol.  ^h^ The points from fruit and vegetable variety, unique fruits and vegetables consumed, red and processed meat, and whole grains were summed and points toward the ACS score were awarded as follows: 0 points awarded for a sum of 0 to 2, 1 point awarded for a sum of 3 to 6, 2 points awarded for a sum of 7 to 9. | | | | |

| **Supplemental Table S2. Individuals Items Included in Each Component of the 2021 Score, 2021 Score + Diet, 2015 Score, ACS Score, ACS Score + Alcohol, and WCRF/AICR Score** | | | | | | | |
| --- | --- | --- | --- | --- | --- | --- | --- |
| **Sub-Component** | **Items** | **2021** | **2021 + Diet** | **2015** | **ACS** | **ACS + Alcohol** | **AICR** |
| **Physical Activity** |  | | | | | | |
| Total | Walking, jogging, running, bicycling, swimming, tennis, squash or racquetball, calisthenics or rowing, other aerobic exercise, weight training | X | X |  |  |  |  |
| Vigorous / Vigorous Aerobic | Walking at a very brisk/striding pace, jogging, running, bicycling, swimming, tennis, squash or racquetball, calisthenics or rowing, other aerobic exercise |  |  | X |  |  | X |
| Moderate or Vigorous | Walking at a normal, brisk, or very brisk/striding pace; bicycling, swimming, tennis, weight training |  |  |  | X |  | X |
| Moderate or Vigorous Aerobic | Walking at a normal, brisk, or very brisk/striding pace; bicycling, swimming, tennis |  |  |  | X | X |  |
| Brisk Walking | Walking at a brisk or very brisk / striding pace |  |  | X |  |  |  |
| Strength Training | any reported weight training |  |  |  | X | X |  |
| **Foods and Beverages** |  | | | | | | |
| Fatty Fish | Dark meat fish, e.g., tuna steak, mackerel, salmon, sardines, bluefish, swordfish |  |  | X |  |  |  |
| Alcohol | Red wine, white wine, regular beer, lite beer, liquor |  |  |  |  | X | X |
| Wine | Red wine, white wine |  | X |  |  |  |  |
| Sugar-Sweetened Beverages | Prune juice, apple juice or cider, orange juice, other fruit juices, Coke or Pepsi or other cola with sugar, other carbonated beverages with sugar, other sugared beverages (punch, lemonade, sports drinks) |  |  |  |  |  | X |
| aUPFs | Non-dairy coffee whitener; sour cream or whipped cream; sherbet or ice milk; ice cream; margarine cottage or ricotta cheese; cream cheese; breaded fish cakes, pieces, or fish sticks; chicken or turkey sandwich or frozen meal; refined cold breakfast cereal; white bread (including pita); English muffins, bagels, or rolls; crackers, triskets, or wheat thins; muffins or biscuits; pancakes or waffles; pancakes or waffles; French fried potatoes; potato chips or corn chips; pizza; tortilla; lo-calorie cola, e.g., tab with caffeine; diet soda without caffeine; dairy coffee drink (cappuccino); pure chocolate candy bar or packet, e.g., Hershey’s, M&M’s; Mixed candy bars, e.g., Snickers, Milky Way, Reese’s; Candy with no chocolate; doughnuts; cake; pie; cookies; sweet rolls, coffee cake, pastry; brownies; breakfast bars, e.g., Nutrigrain, granola, kasha; Energy bars, e.g., Clif, Luna, Glucerna, Powerbar; low carb bars; e.g., Atkins, Zone, South Beach; pretzels; popcorn; chowder or cream soup; red chili sauce; Mayonnaise or other creamy salad dressing; salad dressing; regular mayonnaise; low-fat or fat-free mayonnaise; Splenda; other artificial sweetener; dark bread; mustard. |  |  |  |  |  | X |
| Tomatoes | Tomatoes, tomato juice, tomato sauce, tomato soup, salsa, pizza |  |  | X |  |  |  |
| Whole Fruits and Vegetables (WCRF/AICR) | Raisins or grapes, prunes, bananas, cantaloupe, watermelon, avocado, apples or pears, oranges, grapefruit, strawberries, blueberries, peaches or plums or apricots, tomatoes, tomato juice or V-8, tomato sauce, salsa, celery, string beans, broccoli, cauliflower, cabbage or coleslaw, Brussels sprouts, raw carrots, cooked carrots, mixed or stir fry vegetables or soup, yellow (winter) squash, eggplant or zucchini or other summer squash, kale or mustard greens or chard, raw spinach, cooked spinach, iceberg or head lettuce, romaine or leaf lettuce, alfalfa sprouts, green or yellow or red pepper, onion as a vegetable or rings or soup, onion as a garnish, garlic, beets |  |  |  |  |  | X |
| Whole Fruits and Vegetables (ACS)^a^ | Raisins or grapes, prunes or dried plums, bananas, cantaloupe, watermelon, avocado, apples or pears, oranges, grapefruit, strawberries, blueberries, peaches or plums or apricots, tomatoes, tomato sauce, salsa, celery, string beans, broccoli, cauliflower, cabbage or coleslaw or sauerkraut, Brussels sprouts, raw carrots, cooked carrots, mixed or stir fry vegetables or soup, yellow or orange (winter) squash, eggplant or zucchini or other summer squash, kale or mustard greens or chard, raw spinach, cooked spinach, iceberg or head lettuce, romaine or leaf lettuce, alfalfa sprouts, green or yellow or red pepper, onion as a vegetable or rings or soup, onion as a garnish, beets, yams or sweet potatoes, corn, peas or lima beans fresh or frozen or canned or soup, beans or lentils baked or dried or soup, tofu or soy burger or soybeans or miso or other soy protein |  |  |  | X | X |  |
| Processed Red Meat | Bacon, beef or pork hot dogs, salami or bologna or other processed meat sandwiches, other processed meats (e.g., sausage, kielbasa, etc.) |  | X |  |  |  |  |
| Processed Meat | Bacon, beef or pork hot dogs, salami or bologna or other processed meat sandwiches, other processed meats (e.g., sausage, kielbasa, etc.), chicken or turkey hot dogs or sausage |  |  | X | X | X | X |
| Red Meat | Hamburgers (regular, lean, or extra lean), beef or lamb as a main dish, pork as a main dish, beef or lamb or pork as a sandwich or mixed dish |  | X |  | X | X | X |
| Abbreviations: ACS – American Cancer Society; AICR – American Institute for Cancer Research; aUPF – adapted ultra-processed food; WCRF – World Cancer Research Fund  ^a^ When considering variety of whole fruits and vegetables consumed, each of the following sets contributed only one point to variety, as appropriate: tomatoes and tomato sauce and salsa; raw carrots and cooked carrots; raw spinach and cooked spinach; onions as a vegetable and onions as a garnish | | | | | | | |

| **Supplemental Table S3. Post-diagnostic health behavior scores and the risk of prostate cancer progression among men with non-metastatic prostate cancer, estimated via parametric (Weibull) survival models to account for interval censoring with time zero set to date of diagnosis** | | | | | | | | | | | | | | | |  |  |  |
| --- | --- | --- | --- | --- | --- | --- | --- | --- | --- | --- | --- | --- | --- | --- | --- | --- | --- | --- |
| **2021 Score – HR (95% CI)** | | | | | | | | | | | | | | | |  |  |  |
|  | events | N | Continuous | | | 1^st^ Tertile | 2^nd^ Tertile | | | 3^rd^ Tertile | | | | | P_trend_ | | |  |
|  |  |  |  |  |  | (0-2 pts) | (2.5 pts) | | | (3 pts) | | | | |  | | |  |
| Simple^a^ | 560 | 2,354 | 0.80 | (0.70, | 0.92) | Ref | 0.85 | (0.69, | 1.03) | 0.76 | (0.61, | | 0.95) | | 0.02 | | |  |
| Fully Adjusted^b^ | 407 | 1,870 | 0.82 | (0.69, | 0.97) | Ref | 0.93 | (0.72, | 1.21) | 0.81 | (0.61, | | 1.08) | | 0.16 | | |  |
| **2021 Score + Diet – HR (95% CI)** | | | | | | | | | | | | | | | |  |  |  |
|  | events | N | Continuous | | | 1^st^ Tertile | 2^nd^ Tertile | | | 3^rd^ Tertile | | | | | P_trend_ | | |  |
|  |  |  |  |  |  | (0.25-2.25 pts) | (2.5-3 pts) | | | (3.25-4 pts) | | | | |  |  |  |  |
| Simple^a^ | 560 | 2,354 | 0.82 | (0.72, | 0.93) | Ref | 0.80 | (0.67, | 0.96) | 0.76 | (0.62, | 0.93) | | 0.008 | | |  |  |
| Fully Adjusted^b^ | 424 | 1,940 | 0.85 | (0.73, | 0.99) | Ref | 0.85 | (0.68, | 1.06) | 0.82 | (0.63, | 1.07) | | 0.15 | | |  |  |
| **2015 Score – HR (95% CI)** | | | | | | | | | | | | | | | |  |  |  |
|  | events | N | Continuous | | | 1^st^ Tertile | 2^nd^ Tertile | | | 3^rd^ Tertile | | | | | P_trend_ | | |  |
|  |  |  |  |  |  | (0-3 pts) | (4 pts) | | | (5-6 pts) | | | | |  |  |  |  |
| Simple^a^ | 545 | 2,322 | 0.91 | (0.85, | 0.97) | Ref | 0.86 | (0.66, | 1.11) | 0.79 | (0.64, | 0.99) | | 0.04 | | |  |  |
| Fully Adjusted^b^ | 403 | 1,867 | 0.92 | (0.85, | 1.00) | Ref | 0.86 | (0.66, | 1.11) | 0.81 | (0.61, | 1.07) | | 0.13 | | |  |  |
| **ACS Score – HR (95% CI)** | | | | | | | | | | | | | | | |  |  |  |
|  | events | N | Continuous | | | 1^st^ Tertile | 2^nd^ Tertile | | | 3^rd^ Tertile | | | | | P_trend_ | | |  |
|  |  |  |  |  |  | (0-2 pts) | (2.5-3 pts) | | | (3.5-6 pts) | | | | |  |  |  |  |
| Simple^a^ | 567 | 2,381 | 0.91 | (0.84, | 0.97) | Ref | 0.88 | (0.71, | 1.10) | 0.76 | (0.63, | 0.92) | | 0.005 | | |  |  |
| Fully Adjusted^b^ | 406 | 1,868 | 0.93 | (0.87, | 1.00) | Ref | 1.01 | (0.78, | 1.32) | 0.82 | (0.68, | 0.99) | | 0.04 | | |  |  |
| **ACS Score + Diet – HR (95% CI)** | | | | | | | | | | | | | | | |  |  |  |
|  | events | N | Continuous | | | 1^st^ Tertile | 2^nd^ Tertile | | | 3^rd^ Tertile | | | | | P_trend_ | | |  |
|  |  |  |  |  |  | (0-3.5 pts) | (4-5 pts) | | | (5.5-8 pts) | | | | |  |  |  |  |
| Simple^a^ | 554 | 2,345 | 0.90 | (0.85, | 0.97) | Ref | 0.69 | (0.54, | 0.88) | 0.64 | (0.49, | 0.84) | | 0.002 | | |  |  |
| Fully Adjusted^b^ | 406 | 1,868 | 0.91 | (0.85, | 0.96) | Ref | 0.79 | (0.64, | 0.98) | 0.63 | (0.47, | 0.84) | | 0.001 | | |  |  |
| **WCRF/AICR Score – HR (95% CI)** | | | | | | | | | | | | | | | |  |  |  |
|  | events | N | Continuous | | | 1^st^ Tertile | 2^nd^ Tertile | | | 3^rd^ Tertile | | | | | P_trend_ | | |  |
|  |  |  |  |  |  | (0.75-3.25 pts) | (3.5-4 pts) | | | (4.25-7 pts) | | | | |  |  |  |  |
| Simple^a^ | 569 | 2,380 | 0.91 | (0.81, | 1.02) | Ref | 0.90 | (0.74, | 1.09) | 0.81 | (0.64, | 1.02) | | 0.07 | | |  |  |
| Fully Adjusted^b^ | 410 | 1,876 | 0.90 | (0.79, | 1.02) | Ref | 1.00 | (0.78, | 1.28) | 0.83 | (0.64, | 1.09) | | 0.19 | | |  |  |
| Abbreviations: ACS – American Cancer Society; Adj – adjusted; AICR – American Institute for Cancer Research; CaPSURE – Cancer of the Prostate Strategic Urologic Research Endeavor; CDL – CaPSURE Diet and Lifestyle study; CI – confidence interval; HR – hazard ratio; pts – points; WCRF – World Cancer Research Fund; wk – week  ^a^ Simple models were adjusted for time between diagnosis and date of first CDL questionnaire (continuous) and age at diagnosis (continuous)  ^b^ Fully-adjusted models were additionally adjusted for clinical T stage (T1, T2, T3), Gleason score (<7, 7, >7), diagnostic PSA value (≤6, >6-10, >10-20), primary treatment (radical prostatectomy, active surveillance/watchful waiting, radiotherapy/brachytherapy, hormone therapy, other), family history of prostate cancer in brother of father (yes, no), race (white, non-white), total caloric intake (continuous), plus the following variables (if not part of the score): whole milk intake (≤4 servings/wk, >4 servings/wk), wine intake (3-14 servings/wk, <3 or >14 servings/wk), total alcohol intake (non-drinker, >0-2 serving/day, >2 servings/day), red and processed meat intake (quartiles), tomato intake (continuous), dark meat fish intake (continuous), selenium supplement use (non-user, <140ug/day, ≥140ug/day, user with unknown daily dosage), smoking (never, quit ≥10 years prior, quit <10 years prior, current) | | | | | | | | | | | | | | | | | | |

| **Supplemental Table S4. Post-diagnostic health behavior scores and the risk of prostate cancer progression among 2,056 men with non-metastatic prostate cancer, estimated via using both Cox Proportional Hazards and Fine-Gray methods** | | | | | | | | | | | | | | | |  |  |  |
| --- | --- | --- | --- | --- | --- | --- | --- | --- | --- | --- | --- | --- | --- | --- | --- | --- | --- | --- |
| **2021 Score – HR (95% CI)** | | | | | | | | | | | | | | | |  |  |  |
|  | events | N | Continuous | | | 1^st^ Tertile | 2^nd^ Tertile | | | 3^rd^ Tertile | | | | | P_trend_ | | |  |
|  |  |  |  |  |  | (0-2 pts) | (2.5 pts) | | | (3 pts) | | | | |  | | |  |
| Simple - Cox^a^ | 188 | 1,996 | 0.80 | (0.67 | 0.96) | Ref | 0.77 | (0.58, | 1.04) | 0.69 | (0.46, | | 1.04) | | 0.08 | | |  |
| Simple - FG^a^ | 188 | 1,996 | 0.82 | (0.68 | 0.98) | Ref | 0.80 | (0.60, | 1.06) | 0.71 | (0.47, | | 1.07) | | 0.10 | | |  |
| Fully Adjusted - Cox^b^ | 146 | 1,615 | 0.84 | (0.65 | 1.09) | Ref | 0.91 | (0.63, | 1.32) | 0.79 | (0.50, | | 1.24) | | 0.30 | | |  |
| Fully Adjusted - FG^b^ | 146 | 1,615 | 0.87 | (0.68 | 1.12) | Ref | 0.94 | (0.65, | 1.36) | 0.81 | (0.51, | | 1.28) | | 0.37 | | |  |
| **2021 Score + Diet – HR (95% CI)** | | | | | | | | | | | | | | | |  |  |  |
|  | events | N | Continuous | | | 1^st^ Tertile | 2^nd^ Tertile | | | 3^rd^ Tertile | | | | | P_trend_ | | |  |
|  |  |  |  |  |  | (0.25-2.25 pts) | (2.5-3 pts) | | | (3.25-4 pts) | | | | |  |  |  |  |
| Simple - Cox^a^ | 188 | 1,996 | 0.79 | (0.69, | 0.90) | Ref | 0.84 | (0.67, | 1.04) | 0.67 | (0.46, | 0.97) | | 0.04 | | |  |  |
| Simple - FG^a^ | 188 | 1,996 | 0.81 | (0.70, | 0.92) | Ref | 0.85 | (0.69, | 1.06) | 0.69 | (0.48, | 1.01) | | 0.05 | | |  |  |
| Fully Adjusted - Cox^b^ | 151 | 1,673 | 0.76 | (0.64, | 0.91) | Ref | 0.83 | (0.63, | 1.09) | 0.67 | (0.44, | 1.03) | | 0.07 | | |  |  |
| Fully Adjusted - FG^b^ | 151 | 1,673 | 0.79 | (0.66, | 0.94) | Ref | 0.84 | (0.64, | 1.10) | 0.70 | (0.46, | 1.06) | | 0.09 | | |  |  |
| **2015 Score – HR (95% CI)** | | | | | | | | | | | | | | | |  |  |  |
|  | events | N | Continuous | | | 1^st^ Tertile | 2^nd^ Tertile | | | 3^rd^ Tertile | | | | | P_trend_ | | |  |
|  |  |  |  |  |  | (0-3 pts) | (4 pts) | | | (5-6 pts) | | | | |  |  |  |  |
| Simple - Cox^a^ | 183 | 1,973 | 0.85 | (0.78, | 0.94) | 1.00 | 0.82 | (0.59, | 1.14) | 0.57 | (0.33, | 0.96) | | 0.03 | | |  |  |
| Simple - FG^a^ | 183 | 1,973 | 0.86 | (0.78, | 0.95) | 1.00 | 0.83 | (0.60, | 1.16) | 0.58 | (0.35, | 0.99) | | 0.04 | | |  |  |
| Fully Adjusted - Cox^b^ | 141 | 1,611 | 0.90 | (0.80, | 1.00) | 1.00 | 0.90 | (0.61, | 1.34) | 0.58 | (0.31, | 1.08) | | 0.09 | | |  |  |
| Fully Adjusted - FG^b^ | 141 | 1,611 | 0.91 | (0.82, | 1.02) | 1.00 | 0.92 | (0.62, | 1.37) | 0.60 | (0.32, | 1.11) | | 0.10 | | |  |  |
| **ACS Score – HR (95% CI)** | | | | | | | | | | | | | | | |  |  |  |
|  | events | N | Continuous | | | 1^st^ Tertile | 2^nd^ Tertile | | | 3^rd^ Tertile | | | | | P_trend_ | | |  |
|  |  |  |  |  |  | (0-2 pts) | (2.5-3 pts) | | | (3.5-6 pts) | | | | |  |  |  |  |
| Simple - Cox^a^ | 188 | 2,016 | 0.89 | (0.81, | 0.98) | Ref | 0.94 | (0.68, | 1.29) | 0.71 | (0.53, | 0.95) | | 0.02 | | |  |  |
| Simple - FG^a^ | 188 | 2,016 | 0.90 | (0.81, | 0.99) | Ref | 0.95 | (0.69, | 1.31) | 0.73 | (0.55, | 0.98) | | 0.04 | | |  |  |
| Fully Adjusted - Cox^b^ | 146 | 1,614 | 0.93 | (0.82, | 1.06) | Ref | 1.20 | (0.83, | 1.74) | 0.83 | (0.58, | 1.20) | | 0.32 | | |  |  |
| Fully Adjusted - FG^b^ | 146 | 1,614 | 0.94 | (0.83, | 1.07) | Ref | 1.23 | (0.85, | 1.80) | 0.86 | (0.59, | 1.24) | | 0.41 | | |  |  |
| **ACS Score + Alcohol – HR (95% CI)** | | | | | | | | | | | | | | | |  |  |  |
|  | events | N | Continuous | | | 1^st^ Tertile | 2^nd^ Tertile | | | 3^rd^ Tertile | | | | | P_trend_ | | |  |
|  |  |  |  |  |  | (0-3.5 pts) | (4-5 pts) | | | (5.5-8 pts) | | | | |  |  |  |  |
| Simple - Cox^a^ | 182 | 1,986 | 0.88 | (0.81, | 0.97) | Ref | 0.77 | (0.56, | 1.06) | 0.50 | (0.29, | 0.84) | | 0.009 | | |  |  |
| Simple - FG^a^ | 182 | 1,986 | 0.89 | (0.81, | 0.97) | Ref | 0.78 | (0.57, | 1.07) | 0.51 | (0.30, | 0.85) | | 0.01 | | |  |  |
| Fully Adjusted - Cox^b^ | 146 | 1,614 | 0.89 | (0.81, | 0.99) | Ref | 0.97 | (0.72, | 1.33) | 0.49 | (0.28, | 0.83) | | 0.009 | | |  |  |
| Fully Adjusted - FG^b^ | 146 | 1,614 | 0.90 | (0.81, | 1.00) | Ref | 1.00 | (0.73, | 1.36) | 0.50 | (0.29, | 0.86) | | 0.01 | | |  |  |
| **WCRF/AICR Score – HR (95% CI)** | | | | | | | | | | | | | | | |  |  |  |
|  | events | N | Continuous | | | 1^st^ Tertile | 2^nd^ Tertile | | | 3^rd^ Tertile | | | | | P_trend_ | | |  |
|  |  |  |  |  |  | (0.75-3.25 pts) | (3.5-4 pts) | | | (4.25-7 pts) | | | | |  |  |  |  |
| Simple - Cox^a^ | 188 | 2,013 | 0.86 | (0.73, | 1.01) | Ref | 0.79 | (0.52, | 1.21) | 0.63 | (0.44, | 0.92) | | 0.02 | | |  |  |
| Simple - FG^a^ | 188 | 2,013 | 0.86 | (0.73, | 1.02) | Ref | 0.80 | (0.52, | 1.23) | 0.64 | (0.44, | 0.93) | | 0.02 | | |  |  |
| Fully Adjusted - Cox^b^ | 146 | 1,618 | 0.83 | (0.68, | 1.03) | Ref | 0.90 | (0.51, | 1.56) | 0.61 | (0.37, | 1.02) | | 0.06 | | |  |  |
| Fully Adjusted - FG^b^ | 146 | 1,618 | 0.84 | (0.69, | 1.04) | Ref | 0.90 | (0.52, | 1.56) | 0.62 | (0.37, | 1.03) | | 0.06 | | |  |  |
| Abbreviations: ACS – American Cancer Society; Adj – adjusted; AICR – American Institute for Cancer Research; CI – confidence interval; FG – Fine-Gray; HR – hazard ratio; pts – points; WCRF – World Cancer Research Fund; wk – week  ^a^ Simple models were adjusted for time between diagnosis and date of first CDL questionnaire (continuous) and age at diagnosis (continuous)  ^b^ Fully-adjusted models were additionally adjusted for clinical T stage (T1, T2, T3), Gleason score (<7, 7, >7), diagnostic PSA value (≤6, >6-10, >10-20), primary treatment (radical prostatectomy, active surveillance/watchful waiting, radiotherapy/brachytherapy, hormone therapy, other), family history of prostate cancer in brother of father (yes, no), race (white, non-white), total caloric intake (continuous), plus the following variables (if not part of the score): whole milk intake (≤4 servings/wk, >4 servings/wk), wine intake (3-14 servings/wk, <3 or >14 servings/wk), total alcohol intake (non-drinker, >0-2 serving/day, >2 servings/day), red and processed meat intake (quartiles), tomato intake (continuous), dark meat fish intake (continuous), selenium supplement use (non-user, <140ug/day, ≥140ug/day, user with unknown daily dosage), smoking (never, quit ≥10 years prior, quit <10 years prior, current) | | | | | | | | | | | | | | | | | | |
|  | | | | | | | | | | | | | | | | | | |

| **Supplemental Table S5. Characteristics of men with and without complete data to assess the missing-at-random assumption of multiple imputation** | | | | | | | | | | | |
| --- | --- | --- | --- | --- | --- | --- | --- | --- | --- | --- | --- |
|  | **2021 Score^a^** | | **2015 Score** | | | **ACS Score** | | **ACS Score with Alcohol** | | **WCRF/AICR Score** | |
| **Factor^b^** | Complete | Missing | | Complete | Missing | Complete | Missing | Complete | Missing | Complete | Missing |
| **N** | 1996 | 60 | | 1973 | 83 | 2016 | 40 | 1986 | 70 | 2013 | 43 |
|  |  |  | |  |  |  |  |  |  |  |  |
| **Age (yrs)** | 64.4 (8.0) | 67.3 (7.2) | | 64.3 (7.9) | 67.5 (8.0) | 64.4 (8.0) | 68.7 (6.1) | 64.3 (7.9) | 68.3 (7.7) | 64.4 (7.9) | 67.9 (7.6) |
|  |  |  | |  |  |  |  |  |  |  |  |
| **T-Stage** |  |  | |  |  |  |  |  |  |  |  |
| ≤T1 | 58 | 55 | | 58 | 54 | 57 | 57 | 58 | 53 | 57 | 58 |
| T2 | 41 | 45 | | 41 | 46 | 42 | 42 | 41 | 47 | 42 | 42 |
| T3a | 1 | 0 | | 1 | 0 | 1 | 0 | 1 | 0 | 1 | 0 |
|  |  |  | |  |  |  |  |  |  |  |  |
| **Gleason** |  |  | |  |  |  |  |  |  |  |  |
| <7 | 68 | 58 | | 68 | 60 | 67 | 67 | 67 | 71 | 67 | 60 |
| 7 | 26 | 36 | | 26 | 35 | 26 | 26 | 27 | 22 | 26 | 33 |
| >7 | 6 | 7 | | 6 | 5 | 6 | 8 | 6 | 7 | 6 | 7 |
|  |  |  | |  |  |  |  |  |  |  |  |
| **PSA** | 5.6 [4.4, 7.8] | 6.3 [4.4, 8.3] | | 5.6 [4.4, 7.8] | 6.1 [4.2, 9.6] | 5.6 [4.4, 7.8] | 6.7 [4.9, 8.6] | 5.6 [4.4, 7.8] | 6.3 [4.6, 8.7] | 5.6 [4.4, 7.8] | 6.0 [4.5, 9.3] |
|  |  |  | |  |  |  |  |  |  |  |  |
| **Primary Treatment** |  |  | |  |  |  |  |  |  |  |  |
| Radical Prostatectomy | 63 | 51 | | 63 | 51 | 63 | 38 | 63 | 51 | 63 | 48 |
| AS/WW | 6 | 10 | | 6 | 10 | 6 | 15 | 6 | 12 | 6 | 14 |
| RT/Brachytherapy | 22 | 25 | | 22 | 27 | 22 | 31 | 22 | 22 | 22 | 26 |
| Hormone Therapy | 5 | 8 | | 5 | 10 | 5 | 5 | 5 | 10 | 5 | 5 |
| Other | 4 | 5 | | 4 | 2 | 4 | 10 | 4 | 6 | 4 | 7 |
|  |  |  | |  |  |  |  |  |  |  |  |
| **White race** | 95 | 92 | | 95 | 92 | 95 | 90 | 95 | 89 | 95 | 88 |
|  |  |  | |  |  |  |  |  |  |  |  |
| **Family History of PC** | 20 | 10 | | 20 | 12 | 20 | 8 | 20 | 14 | 20 | 9 |
| Abbreviations: ACS – American Cancer Society; AICR – American Institute for Cancer Research; AS/WW – active surveillance/watchful waiting; IQR – interquartile range; PC – prostate cancer; RT – radiation therapy; SD – standard deviation; WCRF – World Cancer Research Fund  ^a^ The same men were missing the 2021 Score and the 2021 Score with Diet.  ^b^ To ease visual comparisons, summaries are shown as mean (SD), median [IQR], or a percent | | | | | | | | | | | |

| **Supplemental Table S6. Post-diagnostic health behavior scores and the risk of prostate cancer progression among 2,056 men with non-metastatic prostate cancer after multiple imputation to preserve observations with missingness in covariates.** | | | | | |
| --- | --- | --- | --- | --- | --- |
|  | **Continuous^a^** | | | |  |
|  | HR | 95% CI | | p-value |  |
| **2021 Score** | 0.78 | (0.64, | 0.95) | 0.012 |  |
| **2021 Score with Diet** | 0.76 | (0.66, | 0.89) | <0.001 |  |
| **2015 Score** | 0.88 | (0.78, | 0.98) | 0.021 |  |
| **ACS Score** | 0.88 | (0.80, | 0.98) | 0.017 |  |
| **ACS Score with Alcohol** | 0.87 | (0.80, | 0.96) | 0.004 |  |
| **WCRF/AICR Score** | 0.83 | (0.71, | 0.98) | 0.025 |  |
| Abbreviations: ACS – American Cancer Society; Adj – adjusted; AICR – American Institute for Cancer Research; CaPSURE – Cancer of the Prostate Strategic Urologic Research Endeavor; CI – confidence interval; HR – hazard ratio; pts – points; PCSM – prostate cancer specific mortality; WCRF – World Cancer Research Fund; wk – week  ^a^ Multiple imputation resulted in 2,056 complete records and retainment of all 192 progression events.  ^b^ Models were adjusted for clinical CaPSURE site, time between diagnosis and date of first CDL questionnaire (continuous) and age at diagnosis (continuous), clinical T stage (T1, T2, T3), Gleason score (<7, 7, >7), diagnostic PSA value (≤6, >6-10, >10-20), primary treatment (radical prostatectomy, active surveillance/watchful waiting, radiotherapy/brachytherapy, hormone therapy, other), family history of prostate cancer in brother of father (yes, no), race (white, non-white), total caloric intake (continuous), plus the following variables (if not part of the score): whole milk intake (≤4 servings/wk, >4 servings/wk), wine intake (3-14 servings/wk, <3 or >14 servings/wk), total alcohol intake (non-drinker, >0-2 serving/day, >2 servings/day), red and processed meat intake (quartiles), tomato intake (continuous), dark meat fish intake (continuous), selenium supplement use (non-user, <140ug/day, ≥140ug/day, user with unknown daily dosage), smoking (never, quit ≥10 years prior, quit <10 years prior, current). | | | | | |
